# Applying a life course approach to elucidate the biology of sex differences in frailty: early-life gonadectomy diminishes late-life robustness in male and female dogs in the Exceptional Aging in Rottweilers Study

**DOI:** 10.1186/s13293-025-00735-2

**Published:** 2025-07-16

**Authors:** David J. Waters, Aimee H. Maras, Rong Fu, Andres E. Carrillo, Emily C. Chiang, Cheri L. Suckow

**Affiliations:** 1 Center for Exceptional Longevity Studies, Gerald P. Murphy Cancer Foundation, West Lafayette, IN 47906 USA; 2 Center on Aging and the Life Course, Department of Veterinary Clinical Sciences, Purdue University, West Lafayette, IN 47907 USA; 3 Department of Sociology, Siena College, Loudonville, NY 12211 USA; 4 Department of Exercise Science, College of Health Sciences, Chatham University, Pittsburgh, PA 15232 USA

**Keywords:** Preclinical models, Aging, HPG axis, Male-Female differences, Sex hormones, Translational, Neutering, Spay, Companion dogs, Timing of gonadectomy

## Abstract

**Background:**

Frailty refers to a state of increased vulnerability to mortality and other adverse outcomes as a consequence of age-related physiological decline. Sex differences in frailty have been reported; women are usually more frail than men. Physical frailty in men and women is the result of both sociobehavioral and biological factors, making the deciphering of the biology of sex differences in frailty challenging. Investigators have measured frailty in aging animals, including mice and dogs. We posited that companion dogs provide a useful opportunity to study sex differences in the biology of frailty, circumventing many of the sociobehavioral determinants of frailty that complicate human studies.

**Methods:**

Male-female differences in the relationship between lifetime gonad hormone exposure and late-life robustness were studied in the Exceptional Aging in Rottweilers Study (EARS), a lifetime cohort study of companion dogs with a broad range of lifetime gonad exposure. Late-life frailty was assessed by scoring dogs (135 females, 87 males) for deficit accumulation using a 34-item clinical frailty index previously developed and validated in dogs. The study outcome, late-life robustness, was defined as the lowest tertile of frailty index in the study population. Logistic regression models were constructed to assess differences in the likelihood of late-life robustness in dogs stratified into low, middle, and high lifetime gonad exposure groups. Male-female differences were probed after controlling for age at frailty scoring, gonad exposure, and other covariates.

**Results:**

In both male and female dogs, there was a strong association between longer lifetime gonad exposure and increased likelihood of late-life robustness. Compared to dogs in the lowest gonad exposure group, dogs with highest gonad exposure had a statistically significant 3-fold (females) to 10-fold (males) higher likelihood of late-life robustness. Notably, after controlling for gonad exposure and age at frailty scoring, no male-female difference in late-life robustness was found.

**Conclusions:**

The research extends current interest in the biology of sex differences in frailty and provides rationale for further inquiry into the role that the hypothalamic-pituitary-gonadal axis plays in supporting late-life robustness. Studies with companion dogs represent a unique investigative opportunity to enhance our understanding of biological factors that impact sex differences and to spur the development of sex-specific anti-frailty interventions.

**Supplementary Information:**

The online version contains supplementary material available at 10.1186/s13293-025-00735-2.

## Abstract (plain language summary)

In people, “frail” is the descriptor given to individuals with higher vulnerability to adverse future health outcomes. Differences in the degree of frailty versus robustness can be determined by using a clinical tool called frailty index to measure the number of health deficits in each individual. Frailty is diagnosed more often in women than men. Here, we tested in companion dogs whether differences in the occurrence of frailty could be explained by differences in the lifetime duration of sex hormone exposure, i.e., age at gonad removal. We report our experience examining male-female frailty differences in a group of long-lived companion dogs that scientists are utilizing to obtain biological clues on what it takes to achieve extreme longevity. The study outcome, late-life robustness, was defined as those dogs in the study population with the fewest (i.e., lowest tertile) health deficits based on frailty scores. In both males and females, we found a strong association between longer lifetime sex hormone exposure and late-life robustness. Compared to dogs in the shortest sex hormone exposure group, dogs with the longest sex hormone exposure had a statistically significant 3 to 10-fold increased likelihood of late-life robustness, in females and males, respectively. After controlling for differences in sex hormone exposure and age at frailty scoring, no male-female difference in the likelihood of robustness was found. This research indicates the critical role sex hormones may play in retaining late-life robustness and expands our understanding of the importance of early life events in determining lifelong health.

**Supplementary Information:**

The online version contains supplementary material available at 10.1186/s13293-025-00735-2.

## Highlights

Studying the biology of sex differences in frailty in companion dogs can sidestep many of the sociobehavioral determinants of frailty, such as engagement in caregiving, inclination to seek medical help, and smoking behavior, that complicate interpreting frailty differences observed in men and women.

A clinical frailty index that measures deficit accumulation across multiple health domains was employed to assess frailty in geriatric dogs.

In both male and female dogs, a strong association was found between longer lifetime gonadal hormone exposure and increased likelihood of late-life robustness.

After controlling for differences in age and gonad exposure, there was no significant male-female difference in the likelihood of retaining late-life robustness.

This research in companion dogs indicates the critical role sex hormones may play in retaining late-life robustness in both males and females and the value of testing the importance of early life events to spur future discovery of sex-specific, anti-frailty interventions.

**Supplementary Information:**

The online version contains supplementary material available at 10.1186/s13293-025-00735-2.

## Background

Frailty refers to a state of increased vulnerability to mortality and other adverse outcomes as a consequence of age-related decline in physiologic reserve and function [1, 2]. The most widely used frailty assessment tools are frailty phenotype (based on five phenotypic criteria) and frailty index [3]. Frailty index operationalizes frailty as deficit accumulation. By calculating an index representing the proportion of health deficits present in each individual, the FI method can provide valuable insights into the aging process and the heterogeneity of its consequences on mortality risk and other aspects of healthy life expectancy [1, 3–6].

Clear sex differences in frailty have been reported in studies of men and women [7]. Women generally have higher mean frailty index values compared to men; the prevalence of frailty is also higher among women in studies utilizing frailty phenotype [8, 9]. These sex differences are relatively consistent when frailty is quantified using clinical data and self-reports. However, in studies in which frailty index is based upon laboratory test values rather than self-report, the female predominance becomes less consistent, and in some instances men have higher frailty values [9, 10].

Although the mechanisms underlying sex differences in frailty are not well understood, physical frailty in men and women is likely the result of a combination of sociobehavioral and biological factors [11–14]. This entanglement of biological factors with sociobehavioral determinants of frailty, including for example, women being more likely to report health deficits and seek health care [13, 15, 16], makes the deciphering of the biology of sex differences in frailty more challenging. Preclinical studies in mice have afforded the opportunity to explore the biology of sex differences untethered from the complicating effects of these sociobehavioral influences [14]. Considered broadly, results of mouse studies have reinforced the findings in humans – female mice tend to have more frailty than males using clinical data-based measures, but the use of laboratory-based assessments sometimes flips the sex difference to males having higher frailty [17, 18].

By innovating new approaches to probe the biology of sex differences in frailty, comparative biomedical scientists seek to better understand the substantial biological heterogeneity inherent in aging populations. There is scientific interest in harnessing the domesticated dog population as an underutilized opportunity to better understand the genetic and non-genetic determinants of healthy aging [19–25]. Frailty has been measured in dogs by frailty index [26–29] or frailty phenotype [30–33]. But none of these dog studies have focused on sex differences in frailty and none have attempted to dissect out the role that the duration of lifetime gonad exposure plays in the development of frailty in either sex. In men, there is a strong relationship between low serum testosterone and frailty [34, 35], suggesting that degradation of the hypothalamic-pituitary-gonadal (HPG) axis contributes to the development of frailty in older men. In women, younger age at natural menopause is associated with higher likelihood of late-life frailty [36–39], indicating the importance of gonadal hormones and HPG axis integrity. Taken together, the observations point to an important knowledge gap: Could withdrawal of sex hormones earlier in life influence the development of late-life frailty in a sexually dimorphic manner?

In the current study, we set out to address directly a difficulty confronting the field of sex differences in frailty – namely teasing apart the impact of biological factors from sociobehavioral factors. We capitalized on the Exceptional Aging in Rottweilers Study (EARS), a lifetime cohort study of companion dogs with a broad range of lifetime gonad exposure, to uncover the role of sex hormones on late-life frailty in males and females. Living 30% longer than breed average, these dogs represent the canine counterpart of human centenarians. Late-life frailty was captured by scoring dogs for deficit accumulation across numerous health domains using a 34-item clinical frailty index previously developed and validated as a predictor of mortality risk [28]. This canine cohort of 135 females and 87 males enabled an assessment in both sexes of the relationship between gonad exposure and late-life robustness, defined as the lowest tertile of frailty index in the study population. Moreover, we could evaluate sex differences in the biology of physical frailty in a model in which many of the sociobehavioral determinants of frailty in humans, such as engagement in caregiving, inclination to seek medical help, and smoking behavior, were circumvented [7, 14].

Here, we report that, in both male and female dogs, there is a strong association between longer lifetime gonad exposure and increased likelihood of late-life robustness, a result which could not be explained by differences in overweight body condition, reason for gonadectomy, or whether the pet owner reporting frailty was a man or woman. Notably, after controlling for gonad exposure and age at frailty scoring, no significant male-female difference in the likelihood of late-life robustness was found. The research extends current interest in the biology of sex differences in frailty and provides rationale for inquiry into the role of HPG axis integrity in the mechanistic underpinnings of late-life robustness, a gonad-sensitive property of both sexes.

## Methods

### Study sample

The Exceptional Aging in Rottweilers Study (EARS) is an ongoing, ambidirectional cohort of exceptionally long-lived Rottweiler dogs living in the United States and Canada [40]. Dogs enrolled into the study satisfy the following inclusion criteria: (1) validation of purebred status through American Kennel Club (AKC) registry database; (2) age of *≥* 13 years, which represents living at least 30% longer than the average lifespan of the Rottweiler breed [41, 42]; and (3) owner willingness to provide information by questionnaire, medical records, and telephone interviews in order to construct lifetime medical histories. In addition to retrospective data collection, there is a prospective study phase, which includes collection and storage of clinical samples, DNA, and evaluation of tissue samples collected at necropsy. Additional information on enrollment into the EARS cohort has been published elsewhere [43].

Since 2003, more than 400 Rottweilers with exceptional longevity have been enrolled in EARS. Eligible for the current analysis are 222 dogs whose owners agreed to participate in standardized telephone interviews to construct a clinical frailty index (FI). All procedures were implemented in accordance with the Institutional Animal Care and Use Committees of Purdue University and The Gerald P. Murphy Cancer Foundation.

### Assessment of frailty

For each dog, the degree of frailty was measured using a frailty index. The development of this clinical frailty index (EARS-FI) in the Exceptional Aging in Rottweilers Study and its validation as a predictor of mortality have been published [28]. A standardized telephone interview with pet owners conducted by a single interviewer was used to collect responses related to 34 clinical variables in order to score each dog for health deficit accumulation. The interviewer was a veterinarian experienced in canine medicine and age-related decline in physical function of pet dogs. The 34 clinical variables included in EARS-FI satisfied the following criteria: (1) a health deficit that was related to an adverse health status; (2) a deficit which generally increases in prevalence with chronological age; and collectively (3) an array of deficits that reflected perturbation of multiple organ systems; and (4) at least 30 health variables included in the computation [5]. All dogs were alive at the time of frailty assessment and had reached *≥* 13 years of age.

Full details of the 34 items used to construct the clinical frailty index have been previously published [28]. Variables included appetite, strength and stamina, sensory (eyesight, hearing), infection, urinary and fecal continence, sleep, mobility and balance, level of physical activity, mentation, cognition, pain, body condition, hair coat, current disease conditions (endocrine, cardiac, malignant neoplasia), and overall health trajectory. There were no missing data for any of these variables. Most variables were assigned a deficit score of 0 (deficit absent) or 1 (deficit present); for a few variables, scoring of 0.5 was possible if deficit was mild (equivocal) or controlled with medication. For some variables, the scoring of “deficit present” was based upon comparison with the dog’s function as a young adult, i.e. 4 to 6 years old. Frailty index (FI) was calculated as follows: FI equals the sum of health deficits divided by the number of health deficits evaluated. For example, FI calculated for a dog with a total of 14 health deficits would be 14/34, or 0.41.

### Duration of intact hypothalamic-pituitary-gonadal (HPG) axis: age at gonadectomy

Duration of intact HPG axis was defined as duration of lifetime gonad exposure. Duration of lifetime gonad exposure in years was measured by calculating age at gonad removal. In most instances, age at gonadectomy was obtained by comparing date of birth and date of gonad removal surgery found in medical records. When medical record validation of gonad removal date was not available, the date of gonad removal provided in the owner questionnaire was used. If date was recorded in the questionnaire as month/year, the 15th day of the month was used to estimate age at gonadectomy. Twenty-seven males and three females remained intact throughout their lifetime.

### Reason for gonadectomy

In all instances, gonad removal, if performed, was an elective surgical castration or ovariohysterectomy (spay) procedure performed by licensed veterinarians at the discretion of the dog owner, not randomized. A standardized telephone interview was used to collect information to categorize the reason for gonad removal from owners of the females and males that underwent gonad removal: (1) no breeding or no further breeding; (2) treatment or prevention of reproductive or other medical problems (e.g., prostatic disease, mammary gland neoplasia, behavioral issues); (3) substandard conformation (e.g., dental malocclusion, hair color, hip or elbow dysplasia); and (4) treatment of pyometra in females. This interview enabled investigators to segregate dogs into two groups: (1) NO DEFICIT; and (2) PRE-EXISTING DEFICIT representing dogs whose reason for gonad removal was a pre-existing deficit (conformational defect, orthopedic condition such as hip dysplasia, elbow dysplasia or other health conditions such as seizures, pyometra, or prostatitis) that may have potentially influenced lifetime health trajectory, thereby affecting the relationship between gonad removal and the retention of late-life robustness. Even though it is unclear how behavioral issues might influence life-long robustness, dogs were included in the PRE-EXISTING DEFICIT category if a behavioral issue was reported to be the reason for gonadectomy.

### Body condition

Owner-reported body condition of each dog was collected by questionnaire at the time of study entry. Owners were asked to select one of four possible body conditions [underweight; ideal; overweight; markedly overweight (obese)] for three different periods during the life course [pre-adult (6–9 months of age); middle-aged adult (4–6 years of age); and older adult (more than 7 years)]. Owner-reported body condition assessments after 7 years of age were used to group dogs as: (1) overweight; or (2) not overweight.

### Pet owner reporting dog frailty

Because there are reported differences between men and women in health perception and the reporting of symptoms and health deficit accumulation [14, 44, 45], the sex of the dog owner interviewed during dog frailty scoring was also included in the multivariate model. This enabled investigators to determine if this factor, expressed as a binary variable man versus woman reporting dog frailty, significantly influenced the likelihood of late-life robustness in extreme aged dogs.

### Study outcome: late-life robustness

The primary outcome in this study was late-life robustness. In general, robustness refers to the ability to resist deviation from the original state [46]. For the purpose of this study, late-life robustness was defined as those dogs with the lowest third of deficit accumulation measured at extreme age, i.e., lowest tertile of FI values in the study population. Categorizing study subjects based upon data spread (e.g. medians, tertiles, quartiles) have been employed in human studies that used frailty index [47–50]. Choosing this method enabled the evaluation of factors in each sex that were underrepresented or overrepresented in the subset of extreme aged dogs that had the lowest deficit accumulation. In this study population, the relevance of categorizing dogs with lowest tertile of deficit accumulation into a late-life robustness group was verified by showing that dogs in the late-life robustness group had significantly lower mortality risk than dogs with higher FI values. After setting dogs with higher frailty index as the reference group (odds ratio (OR) = 1.0), age-adjusted risk for mortality in dogs with lowest tertile of FI values was 57–76% lower [age-adjusted OR_2 − months mortality_ (95%CI) = 0.24(0.08-0.72) (*p* = .01) and age-adjusted OR_6 − months mortality_ (95%CI) = 0.43(0.23-0.80) (*p* = .01)].

### Data analysis

Descriptive characteristics of the overall study cohort, males only, and females only were expressed as medians and interquartile range (IQR) or range, or proportions (%) and compared using Chi-square and independent-samples median tests. Frequency distribution plots were created to visually display the heterogeneity of FI values in the study cohort and age at frailty scoring. Estimated frailty limit was defined as 99% of FI values [51].

Logistic regression was used to estimate the association between differences in lifetime gonad exposure and likelihood of late-life robustness. In all analyses, late-life robustness was defined as an FI value at the time of frailty scoring in the lowest tertile of FI values for the entire study sample, i.e., lowest population tertile. To estimate the gonad effect on late-life robustness, a logistic regression model was used to generate age-adjusted odds ratios (OR) and 95% confidence intervals (95% CI). For males, age-adjusted ORs were calculated for three subgroups differing in the duration of lifetime testis exposure: (1) less than 2 years (shortest duration of intact HPG axis) (*n* = 20); (2) 2.0–9.8 years (*n* = 34); and (3) > 9.8 years (longest duration of intact HPG axis) (*n* = 33). For females, age-adjusted ORs were calculated for three subgroups differing in the duration of lifetime ovary exposure: (1) less than 2 years (shortest duration of intact HPG axis) (*n* = 40); (2) 2.0–5.5 years (*n* = 49); and (3) > 5.5 years (longest duration of intact HPG axis) (*n* = 46). For each sex, the method used to establish cutpoints segregated dogs that had not retained gonads throughout the developmental period (i.e., the first two years of life) and then equally divided the remaining dogs into middle gonad exposure and highest gonad exposure groups. In each model, the subgroup with low gonad exposure served as the reference group (OR = 1.0). Body condition (overweight versus not overweight), birth cohort (earlier versus later), pet owner reporting frailty (man versus woman) were also included in the logistic regression model as covariates. In 218 dogs for which data on reason for gonad removal were available, likelihood of late-life robustness was evaluated after adjusting for reason for gonad removal expressed as a binary variable (pre-existing deficit versus all other reasons for gonad removal). To assess the robustness of the main findings, adjusted ORs were also compared between males and females using the same gonad exposure cutpoint of 5.5 years for both sexes.

To probe for sex differences in the likelihood of late-life robustness, logistic regression was used to estimate male-female differences in robustness in dogs stratified by lifetime gonad exposure into low, middle, and high exposure groups. In this analysis, males and females in the low gonad exposure group were combined (*n* = 60; 20 males, 40 females) and then age-adjusted OR and 95%CI for robustness were calculated for females, using males as the ref group (OR = 1.0). This approach was repeated to generate age-adjusted ORs for robustness in the middle gonad exposure group (*n* = 83; 33 males, 50 females) and high gonad exposure group (*n* = 79; 34 males, 45 females) using sex-specific cutpoints (5.5 years for females, 9.8 years for males). Sex differences in late-life robustness were then probed in a multivariate model including sex and treating lifetime gonad exposure as a continuous variable (years) in the 222 dogs overall and in the 218 dogs in which reason for gonadectomy was available.

Data analyses were performed using STATA Version 17 (Stata Corp., College Station, TX, USA). Statistical significance was defined as *p* <.05 and all tests were two sided.

## Results

### Description of study cohort

The study cohort consisted of 222 purebred Rottweiler dogs in the Exceptional Aging in Rottweilers Study (EARS) that underwent standardized assessment of frailty using a clinical frailty index (EARS-FI) (Table 1). The 222 dogs in this cohort lived in 209 households in 43 U.S states and Canada. Median (IQR) age at frailty scoring was 13.3 (13.1–13.7) years (Supplementary Fig. 1). Females outnumbered males in the analytic sample: 135 females and 87 males were evaluated. Three of the females and 27 males had intact gonads at time of frailty scoring. Median (IQR) duration of intact HPG axis was 4.6 (1.7–8.0) years. Thirty-nine of 222 (18%) dogs were considered overweight after seven years of age, based upon owner questionnaire responses. Frailty scoring by telephone interview occurred more often with dog owners who were women (*n* = 192) than men (*n* = 30).

The most common reason for gonadectomy was finished breeding or no intention to breed (34 males, 85 females). Reason for gonadectomy was available for 218 dogs and attributed to pre-existing health-related deficits in 60 of 188 (32%) dogs that underwent gonad removal. Among 17 males in the pre-existing deficit as reason for gonadectomy group, the deficits were hip dysplasia/elbow dysplasia (*n* = 12), seizures (*n* = 2), cranial cruciate ligament rupture (*n* = 2), and cryptorchidism (*n* = 1). Among 43 females in the pre-existing deficit as reason for gonadectomy group, the deficits were pyometra (*n* = 16), hip dysplasia/elbow dysplasia (*n* = 10), behavioral issues (*n* = 5), mammary tumor (*n* = 3), seizures (*n* = 2), benign vaginal/uterine lesions (*n* = 2), cranial cruciate ligament rupture (*n* = 2), abnormal gait (*n* = 1), reproductive failure (*n* = 1), and subaortic stenosis (*n* = 1).

**Table 1:** Summary of characteristics of 222 dogs from the Exceptional Aging in Rottweilers Study

Variable	Total (*n* = 222)	Females (*n* = 135)	Males (*n* = 87)	*p*-value
Residence				
Number of U.S. States	43 states and Canada	39 states and Canada	28 states and Canada	--
Number of households	209	131	85	--
Duration of Lifetime Gonad Exposure, median (IQR), in years	4.6 (1.7, 8.0)	3.8 (1.4, 6.6)	6.5 (2.0, 13.4)	< 0.001*
Frailty Index, median (range)	0.44 (0.18, 0.68)	0.44 (0.18, 0.68)	0.44 (0.20, 0.65)	0.84
Age at Frailty Scoring, median (IQR), in years	13.3 (13.1, 13.7)	13.3 (13.1, 13.8)	13.3 (13.2, 13.6)	0.87
Age at Death, median (IQR), in years	14.0 (13.6, 14.6)	14.1 (13.7, 14.7)	14.0 (13.6, 14.4)	0.17
Overweight Body Condition after 7 years of age, n (%)	39 (18)	26 (19)	13 (15)	0.41
Reason for Gonad Removal				
Intact, n (%)	30 (14)	3 (2)	27 (31)	--
Pre-existing health deficit, n (%)**	60 (32)	43 (33)	17 (30)	0.685

Mann-Whitney U test was used to assess sex differences in Duration of Lifetime Gonad Exposure, Frailty Index, Age at Frailty Scoring, and Age at Death. IQR = interquartile range; n = number of dogs

* Indicates a statistically significant difference between females and males

** Percentage value indicates number of dogs within the Reason for Gonad Removal category divided by the total number of dogs that underwent gonad removal for which Reason for Gonad Removal was available (*n* = 188 dogs; 43 of 131 females, 17 of 57 males). Reason for Gonad Removal was not available for one female and three males in the study cohort

### Heterogeneity of frailty in dogs assessed by a clinical frailty index

**Fig. 1:**
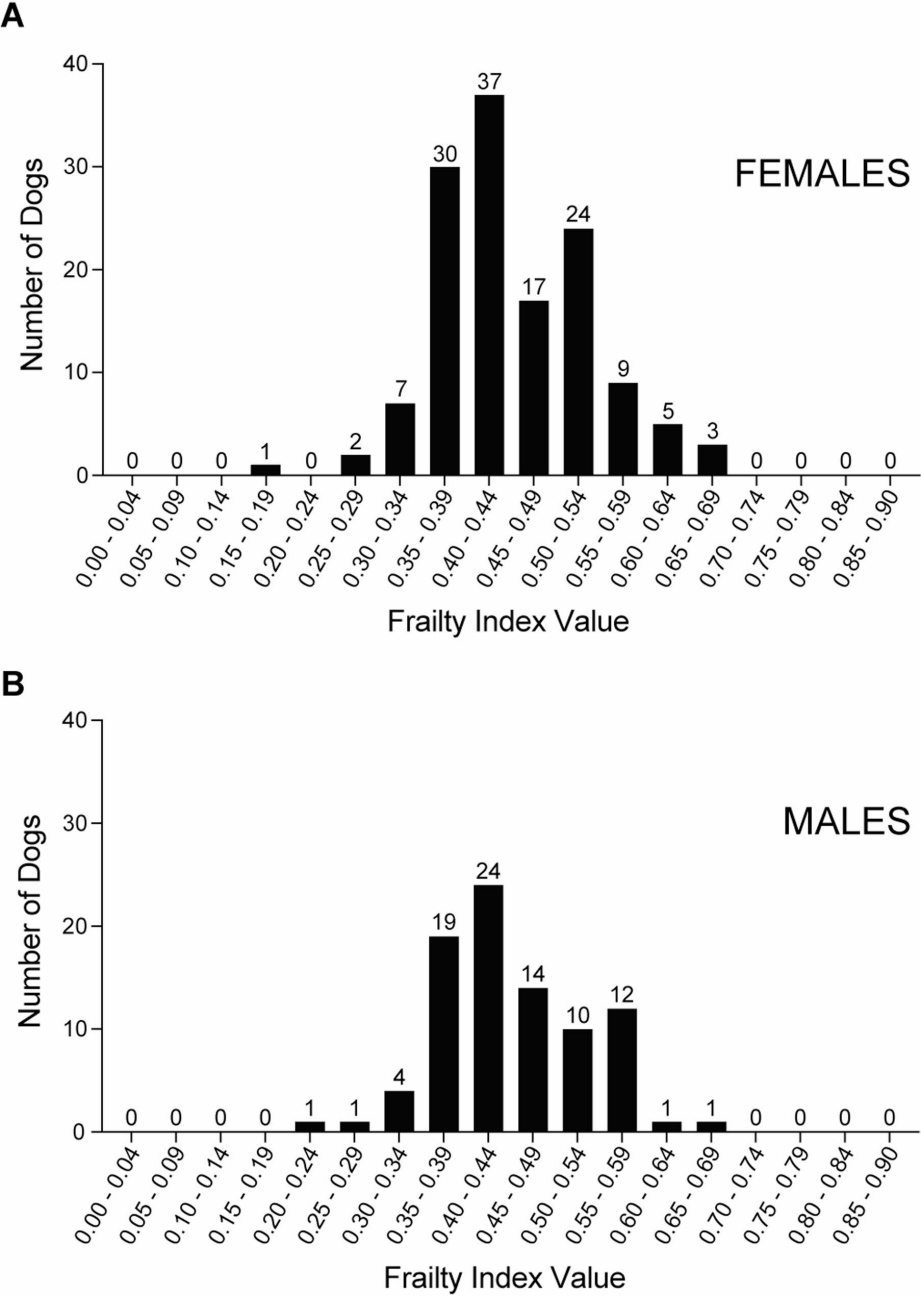
Distribution of frailty index values in 222 dogs from the Exceptional Aging in Rottweilers Study. **(A)** Females (*n* = 135). **(B)** Males (*n* = 87)

The distribution of frailty index values illustrated in Fig. 1 shows considerable heterogeneity in the extent of frailty among dogs reaching extreme longevity. Median (IQR) of FI values was 0.44 (0.38 – 0.50). No dogs had FI < 0.18. Estimated frailty limit, defined as 99th percentile of FI values, was 0.65.

### Higher likelihood of late-life robustness in males and females is associated with longer lifetime gonad exposure

**Fig. 2:**
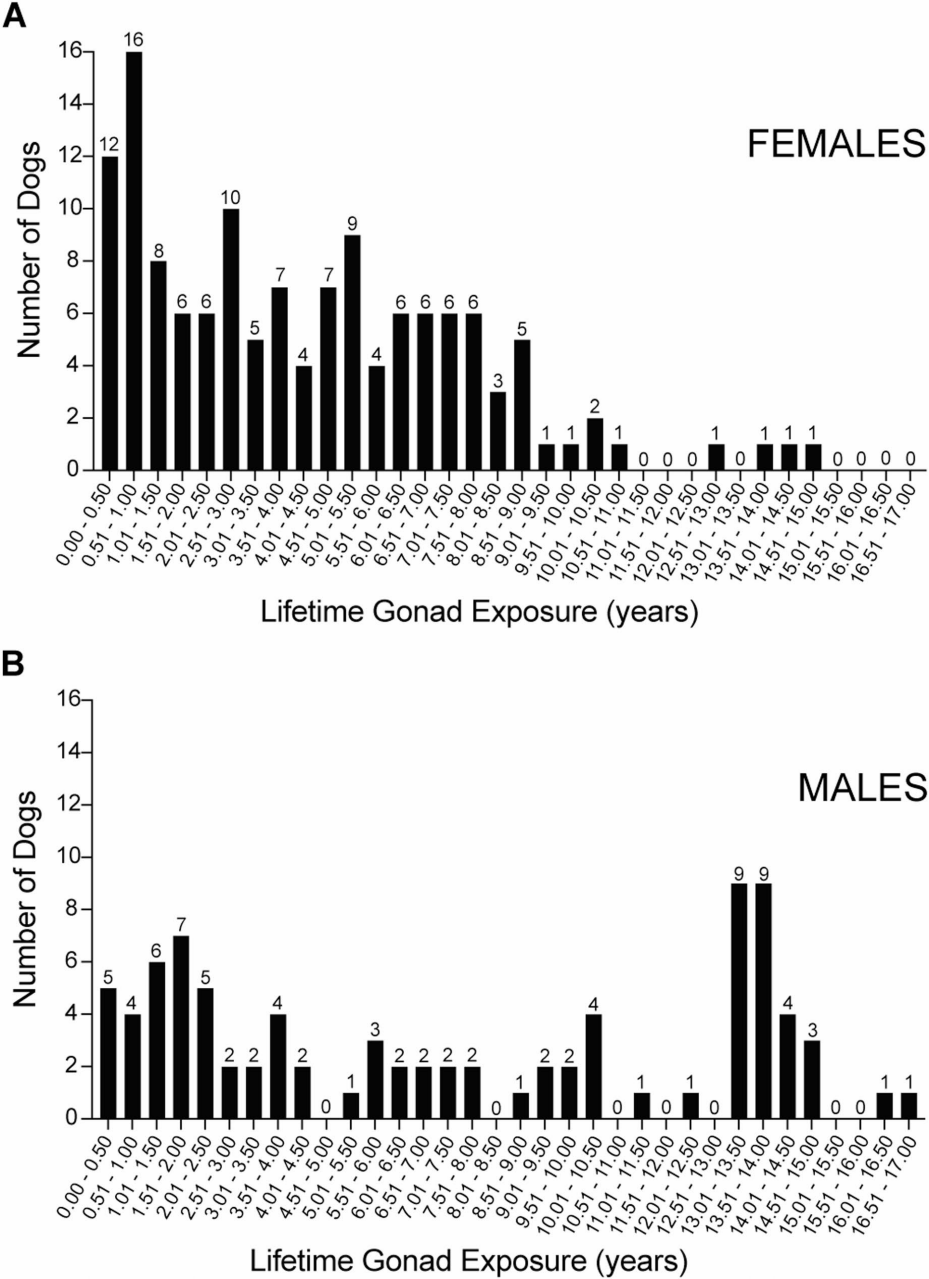
Distribution of duration of lifetime gonad exposure in 222 dogs from the Exceptional Aging in Rottweilers Study. **(A)** Females (*n* = 135). **(B)** Males (*n* = 87)

Males and females in this cohort showed a broad range of lifetime gonad exposure (range, 0.3 to 16.5 years and 0.4 to 14.7 years in males and females, respectively) (Fig. 2). To test the potential impact of differences in lifetime gonad exposure on the likelihood of late-life robustness, age-adjusted ORs were calculated for low, middle and high gonad exposure subgroups. In males, the association between longer lifetime gonad exposure and retaining robustness was particularly strong. Compared to males in the low gonad exposure group (gonadectomy prior to 2 years of age), males with the longest gonad exposure were more than 13 times more likely to have late-life robustness [OR_age−adjusted_ = 13.30 (95%CI, 1.59–111.21) (*p* = .02)] (Table 2). In females, there was also a significant association between longer gonad exposure and late-life robustness. Compared to females in the low gonad exposure group (gonadectomy prior to 2 years of age), females with the longest gonad exposure were almost 3 times more likely to have late-life robustness [OR_age−adjusted_ = 2.86 (95%CI, 1.04–7.89) (*p* = .04)] (Table 3). In multivariate logistic regression, including age at frailty scoring, body condition, birth cohort, and man or woman reporting dog frailty scores, these relationships remained intact. Males with the longest gonad exposure were 10.75 times more likely to have late-life robustness (*p* = .03) (Table 2), while females with the longest gonad exposure were 3.17 times more likely to have late-life robustness (*p* = .04) (Table 3). Younger age at frailty scoring in females, but not in males, was associated with higher likelihood of robustness. There was no significant association between robustness and overweight body condition, early/late birth cohort, or male/female owner reporting frailty. Using a cutpoint of 5.5 years between middle and high gonad exposure groups in both males and females yielded similar results (compare Table 2 with Supplementary Table 1). Taken together, it is concluded that retention of late-life robustness is gonad-sensitive in both sexes.

**Table 2:** Unadjusted and adjusted odds ratios (OR) for likelihood of late-life robustness associated with duration of lifetime gonad exposure and other risk variables in 87 male dogs

	Unadjusted OR (95% CI)	*p*-value	Adjusted OR (95% CI)	*p*-value
Age at Frailty Scoring	0.99 (0.92–1.07)	0.84	1.02 (0.94–1.10)	0.67
Duration of Lifetime Gonad Exposure				
< 2 years	1.0 (ref)		1.0 (ref)	
2.0–9.8 years	8.26 (0.97–70.46)	0.054	6.18 (0.69–55.5)	0.10
> 9.8 years	13.30 (1.59-111.21)	0.02	10.75 (1.23–93.99)	0.03
Body Condition				
Not overweight	1.0 (ref)		1.0 (ref)	
Overweight	0.71 (0.18–2.83)	0.63	0.71 (0.16–3.21)	0.65
Birth Cohort				
Early	1.0 (ref)		1.0 (ref)	
Late	1.68 (0.65–4.30)	0.28	2.04 (0.72–5.74)	0.18
Pet Owner Reporting Dog Frailty				
Woman	1.0 (ref)		1.0 (ref)	
Man	0.27 (0.06–1.29)	0.10	0.40 (0.07–2.20)	0.29

Estimated likelihood of late-life robustness in males associated with duration of gonad exposure (i.e., duration of intact HPG axis) and other exposure variables generated using bivariate logistic regression is shown as an unadjusted odds ratio (OR) and 95% confidence interval (95%CI). Adjusted ORs are calculated for each variable with all five variables in multivariate analysis. Age at frailty scoring was treated as a continuous variable (years). To assess duration of intact HPG axis, male dogs were segregated into three different gonad exposure groups based on age at gonadectomy (see text). Overweight body condition refers to overweight after seven years of age based on owner report. Late-life robustness was defined as frailty index values within the lowest tertile of the study population (*n* = 222). ref = reference group

**Table 3:** Unadjusted and adjusted odds ratios (OR) for likelihood of late-life robustness associated with duration of lifetime gonad exposure and other risk variables in 135 female dogs

	Unadjusted OR (95% CI)	*p*-value	Adjusted OR (95% CI)	*p*-value
Age at Frailty Scoring	0.88 (0.80–0.96)	0.005	0.87 (0.80–0.96)	0.004
Duration of Lifetime Gonad Exposure				
< 2 years	1.0 (ref)		1.0 (ref)	
2.0–5.5 years	2.22 (0.81–6.09)	0.12	2.20 (0.76–6.36)	0.15
> 5.5 years	2.86 (1.04–7.89)	0.04	3.17 (1.08–9.34)	0.04
Body Condition				
Not overweight	1.0 (ref)		1.0 (ref)	
Overweight	0.50 (0.18–1.45)	0.20	0.51 (0.16–1.59)	0.25
Birth Cohort				
Early	1.0 (ref)		1.0 (ref)	
Late	0.60 (0.26–1.37)	0.22	0.54 (0.22–1.33)	0.18
Pet Owner Reporting Dog Frailty				
Woman	1.0 (ref)		1.0 (ref)	
Man	0.40 (0.08–1.90)	0.25	0.38 (0.07–1.98)	0.25

Estimated likelihood of late-life robustness in females associated with duration of gonad exposure (i.e., duration of intact HPG axis) and other exposure variables generated using bivariate logistic regression is shown as an unadjusted odds ratio (OR) and 95% confidence interval (95%CI). Adjusted ORs are calculated for each variable with all five variables in multivariate analysis. Age at frailty scoring was treated as a continuous variable (years). To assess duration of intact HPG axis, females were segregated into three different gonad exposure groups based on age at gonadectomy (see text). Overweight body condition refers to overweight after seven years of age based on owner report. Late-life robustness was defined as frailty index values within the lowest tertile of the study population (*n* = 222). ref = reference group

Because the reason for gonadectomy in this observational cohort might influence age at gonadectomy and lifetime health trajectories, we evaluated the association between reason for gonadectomy and late-life robustness. Neither males nor females with pre-existing health deficits as reason for gonadectomy had a significantly reduced likelihood of robustness compared to dogs in which gonadectomy was not prompted by health deficits. Compared to dogs in the group without pre-existing health deficits, age-adjusted ORs for late-life robustness in dogs with pre-existing deficits as reason for gonadectomy were 0.72 (0.21–2.49) (*p* = .61) and 0.995 (0.44–2.26) (*p* = .99) in males and females, respectively (Table 4). Further, reason for gonadectomy did not attenuate the strong association between longer gonad exposure and late-life robustness observed in both sexes (Supplementary Tables 2 and 3). Taken together, these results confirm that late-life robustness is a gonad-sensitive property that is not significantly affected by the reason for gonad removal.

**Table 4:** Unadjusted and age-adjusted odds ratios (OR) for the likelihood of late-life robustness in male and female dogs with pre-existing health deficits reported as reason for gonadectomy

	Reason for Gonadectomy	Unadjusted OR (95% CI)	*p*-value	Age-Adjusted OR (95% CI)	*p*-value
Males (*n* = 84)	No Deficit	1.0 (ref)		1.0 (ref)	
	Deficit	0.72 (0.21–2.49)	0.61	0.72 (0.21–2.49)	0.61
Females (*n* = 134)	No Deficit	1.0 (ref)		1.0 (ref)	
	Deficit	1.03 (0.47–2.27)	0.95	0.995 (0.44–2.26)	0.99
Combined (*n* = 218)	No Deficit	1.0 (ref)		1.0 (ref)	
	Deficit	0.93 (0.48–1.80)	0.84	0.93 (0.48–1.80)	0.82

Late-life robustness defined as frailty index values within the lowest tertile of the study population. Unadjusted and age-adjusted odds ratios (OR) and 95% confidence interval (95%CI) calculated using logistic regression. Dogs were dichotomized into two groups on the basis of reason for gonadectomy: pre-existing health deficit (Deficit) or other reasons for gonadectomy (No Deficit). Odds ratio for group with pre-existing deficit as reason for gonadectomy are reported, with the no deficits group serving as reference (ref) group (OR = 1.0)

### Likelihood of late-life robustness is not significantly different between males and females after adjusting for differences in lifetime gonad exposure and age at frailty scoring

Median (range) FI value in males [0.44 (0.20-0.65)] and females [0.44 (0.18-0.68)] did not differ significantly (*p* = .84) (Table 1). The proportion of dogs that retained late-life robustness was 25 of 87 (29%) males compared with 40 of 135 (30%) females (*p* = .89). There was no significant difference in age at frailty scoring between males and females (*p* = .87) (Table 1). Importantly, the likelihood of retaining robustness did not significantly differ between males and females after adjusting for lifetime gonad exposure and age at frailty scoring (Table 5). Age-adjusted likelihood of late-life robustness for females compared to males in the low, middle, and high gonad exposure groups was 3.91 (*p* = .22), 1.09 (*p* = .87), and 0.96 (*p* = .93), respectively (Table 5). Using 5.5 years as cutpoint between middle and high gonad exposure groups in both sexes yielded similar results (compare Table 5 with Supplementary Table 4). In the overall model, significant sex differences in the likelihood of late-life robustness were not identified (Table 6). In multivariate analysis including age at frailty scoring, lifetime gonad exposure, body condition, birth cohort, or whether owner interviewed for dog frailty scoring was a man or woman, the adjusted OR for late-life robustness in females was 1.69 (0.82–3.46) (*p* = .15), compared to males (Table 6). Though sex-differences in robustness were not detected, the association between gonad exposure and robustness remained strong in the combined model, with a 14% increase in likelihood of late-life robustness for each 1-year increase in lifetime gonad exposure (OR_adjusted_ = 1.14 (1.06–1.24) (*p* = .001) (Table 6). Including reason for gonadectomy in the model did not alter these main findings (Table 6). Overall, the results indicate retention of late-life robustness is sensitive to differences in the duration of gonad exposure throughout the life course. The results, however, suggest that after controlling for lifetime gonad exposure, there were no significant sex differences in the likelihood of geriatric dogs retaining robustness.

**Table 5:** Comparison of male-female differences in unadjusted and age-adjusted odds ratios (OR) for the likelihood of late-life robustness in 222 dogs stratified into three categories of lifetime gonad exposure

Lifetime Gonad Exposure Category	Sex	Unadjusted OR (95% CI)	*p*-value	Age-Adjusted OR (95% CI)	*p*-value
Low* *n* = 60	Males	1.0 (ref)		1.0 (ref)	
	Females	4.03 (0.46–35.30)	0.21	3.91 (0.44–34.58)	0.22
Middle** *n* = 83	Males	1.0 (ref)		1.0 (ref)	
	Females	1.08 (0.42–2.80)	0.87	1.09 (0.42–2.81)	0.87
High*** *n* = 79	Males	1.0 (ref)		1.0 (ref)	
	Females	0.87 (0.35–2.16)	0.76	0.96 (0.37–2.48)	0.93

Late-life robustness defined as frailty index values within the lowest tertile of the study population (*n* = 222). Unadjusted and age-adjusted odds ratios (OR) and 95% confidence interval (95%CI) for each lifetime gonad exposure category calculated using logistic regression. Males serve as the reference (ref) group (OR = 1.0) for each gonad exposure category.

*Low lifetime gonad exposure group consisting of 20 males with exposure < 2 years, 40 females with exposure < 2 years

**Middle lifetime gonad exposure group consisting of 34 males with exposure 2–9.8 years, 49 females with exposure 2–5.5 years

***High lifetime gonad exposure group consisting of 33 males with exposure > 9.8 years, 46 females with exposure > 5.5 years

**Table 6:** Multivariate risk model of likelihood of late-life robustness: Male-female differences in 222 dogs and in subcohort of 218 dogs with reason for gonadectomy

	Multivariate OR (95% CI) *n* = 222	*p*-value	Multivariate OR (95% CI) *n* = 218	*p*-value
Age at Frailty Scoring	0.94 (0.88–0.99)	0.02	0.93 (0.88–0.99)	0.01
Sex				
Male	1.0 (ref)		1.0 (ref)	
Female	1.69 (0.82–3.46)	0.15	1.88 (0.90–3.96)	0.10
Duration of Lifetime Gonad Exposure (years)	1.14 (1.06–1.24)	0.001	1.15 (1.06–1.25)	0.001
Body Condition				
Not overweight	1.0 (ref)		1.0 (ref)	
Overweight	0.47 (0.19–1.16)	0.10	0.49 (0.19–1.22)	0.13
Birth Cohort				
Early	1.0 (ref)		1.0 (ref)	
Late	0.92 (0.48–1.77)	0.81	0.98 (0.51–1.90)	0.95
Pet Owner Reporting Dog Frailty				
Woman	1.0 (ref)		1.0 (ref)	
Man	0.43 (0.14–1.36)	0.15	0.46 (0.14–1.45)	0.18
Reason for Gonadectomy				
No Deficit	–		1.0 (ref)	
Deficit	–		0.89 (0.43–1.80)	0.74

Estimated likelihood of late-life robustness associated with duration of gonad exposure (i.e., duration of intact HPG axis) and other exposure variables generated using multivariate logistic regression is shown as odds ratio (OR) and 95% confidence interval (95%CI). Multivariate ORs are calculated for each variable with all five variables in multivariate model. To evaluate male-female differences, ORs for likelihood of late-life robustness are reported for females, with males serving as the reference (ref) group (OR = 1.0). Age at frailty scoring and duration of lifetime gonad exposure were treated as continuous variables (years). Overweight body condition refers to overweight after seven years of age based on owner report. Late-life robustness was defined as frailty index values within the lowest tertile of the study population. In the right column of the table, multivariate analysis is presented for the subcohort of 218 dogs for which reason for gonadectomy was available. These dogs were dichotomized into two groups on the basis of reason for gonadectomy: pre-existing health deficit (Deficit) or other reasons for gonadectomy (No Deficit). Odds ratio for the group with pre-existing Deficit as reason for gonadectomy are reported, with the No Deficit group as reference (ref) group

## Discussion

Here, we set out to directly address one of the challenges facing the field of sex differences in frailty – teasing apart the impact of biological factors from sociobehavioral factors affecting late-life robustness. First, we investigated the extent to which late-life robustness is a property that is sensitive to differences in the duration of lifetime gonad exposure. The results were obtained by scoring a cohort of geriatric dogs for late-life robustness, defined as the lowest tertile of frailty index in the study population. This canine cohort of 135 females and 87 males had a broad range of lifetime gonad exposure, which not only enabled a rigorous assessment of the relationship between gonad exposure and late-life robustness in both sexes, but also allowed the pursuit of sex differences in the biology of frailty without being complicated by many of the sociobehavioral factors that impact human frailty. In both male and female dogs, we found a strong association between longer gonad exposure and increased likelihood of late-life robustness. Compared to individuals in the lowest gonad exposure group (less than 2 years), those with the highest gonad exposure had a statistically significant 3-fold (in females) to 10-fold (in males) higher likelihood of late-life robustness. The robustness in males and in females associated with longer gonad exposure could not be explained by differences in overweight body condition, birth cohort, or whether the pet owner reporting frailty was a man or woman. Because age at gonadectomy was not randomized in this observational study, standardized information on reason for gonadectomy was collected from interviews with dog owners. Importantly, we found no association between the reason for gonadectomy and likelihood of late-life robustness. Moreover, the relationship between longer gonad exposure and increased robustness was not attenuated by whether reason for gonadectomy was attributed to pre-existing health deficits. Next, we investigated whether there were sex differences in late-life robustness in this study cohort. After controlling for duration of gonad exposure and age at frailty scoring, we found no male-female difference in robustness. An analysis of age-adjusted odds of late-life robustness within three different strata of gonad exposure, along with multivariate modeling of the entire study cohort including reason for gonadectomy and with gonad exposure treated as a continuous variable, failed to demonstrate any significant male-female difference in late-life robustness. The research extends current interest in the biology of sex differences in frailty, spurring further inquiry into how HPG axis integrity contributes to late-life robustness, which is gonad-sensitive in both sexes. Moreover, the results justify future studies capitalizing on this and other canine lifetime cohorts to apply a life-course approach to the study of later-life frailty. By re-envisioning studies with companion dogs as a unique, large animal preclinical model, we may move closer to understanding the biological factors that impact sex differences in frailty, thereby advancing the development of sex-specific anti-frailty interventions.

Frailty index (FI) operationalizes frailty as health deficit accumulation and has been used to expose hidden heterogeneity in aging populations. Older adults with higher FI have higher vulnerability to mortality and a range of other adverse outcomes, including falling, prolonged hospitalization stay, and incident dementia [2, 52]. Earlier, we developed a clinical frailty index (EARS-FI) constructed using information collected from standardized telephone interviews with dog owners, and then validated this clinical FI as a predictor of all-cause mortality in 93 extreme aged dogs of the EARS study [28]. Here, we used this clinical FI to capture deficit accumulation across multiple domains of health in 222 extreme aged dogs in order to test the relationship between lifetime gonad exposure and late-life robustness in both sexes.

In the current study, we defined late-life robustness as the lowest tertile of frailty index in the study population (FI *≤* 0.38). In the human literature, considerable variation exists in the categorization of FI values, with cutpoints for frail versus non-frail ranging from FI of 0.12 to 0.45 [53], with FI = 0.25 the most frequently used overall, and FI = 0.35 most commonly used in oncology studies [54]. The purpose of our study was not to establish a cutpoint to report the prevalence of frailty in this or any other study population, or to develop standardized clinical interventions on the basis of any particular FI cutpoint. Instead, we sought to evaluate factors in each sex that segregated with lowest deficit accumulation at extreme age. We established late-life robustness as dogs with FI *≤* 0.38 after examining the spread of the FI values in our analytic sample and then categorized robustness as frailty values in the lowest population tertile. Categorization methods based upon data spread (e.g. medians, quartiles) have been used in previous human studies that measured frailty index [47–50]. Importantly, our method provided sufficient number of dogs in the late-life robustness subgroup to evaluate the relationship between gonad exposure and lower deficit accumulation. In contrast, the use of a more severe cutpoint of FI < 0.25 for robustness would have held no utility in our study, since only two of the 222 extreme aged dogs had FI < 0.25. Further justification that the categorizing of frailty index used here provided a relevant outcome measure in this study population was obtained by demonstrating that dogs included in the late-life robustness group had significantly lower mortality risk than dogs with higher FI values (see Methods).

It has been postulated that degradation of the HPG axis contributes to the development of frailty in older men and women [34, 35, 55]. In both sexes, the secretion of sex hormones by the gonad are under the control of hypothalamic gonadotropin releasing hormone and pituitary gonadotropin secretion. In human studies, assessment of HPG axis integrity is often expressed as serum level of gonadal hormones. In men, this means evaluating the association between serum testosterone levels and frailty, although elevated levels of the gonadotropin luteinizing hormone (LH), independent of serum testosterone levels, have been associated with higher frailty in some studies [56, 57]. In a 2023 meta-analysis [34], a strong association between low serum testosterone (total, free) and frailty phenotype was found in seven of seven cross-sectional studies evaluated [58–64]. Longitudinal studies suggest lower baseline testosterone levels can also predict increase in frailty during the ensuing 12–24 months [65–67], though this relationship was not confirmed in other studies [59, 68, 69]. In contrast to these findings in men, a single study randomizing 21 male C57BL6 mice to gonadectomy versus sham surgery showed gonadectomized mice did not have higher frailty scores [70]. In studies of women, age at oophorectomy or natural menopause are frequently used to measure duration of HPG axis integrity. Loss of ovarian hormones secondary to bilateral oophorectomy in premenopausal women is linked to alterations in fundamental aging processes, leading to frailty and multimorbidity [71]. Older age at natural menopause, i.e. longer duration of lifetime gonad hormone exposure, is associated with lower likelihood of frailty later in life [36, 38, 39]. The results presented in the current report provide clear support in both sexes for the notion that late-life frailty is gonad-sensitive. We reached these conclusions by applying a life course approach, using a method that enabled us to report precisely the lifetime duration of HPG axis integrity in each individual, rather than relying upon serum hormone levels to detect hypogonadism, which may be subject to differences in interpretation.

Frailty has been measured in companion dogs in eight previous studies. Four studies used frailty index [26–29], four studies used frailty phenotype [30–33], and the median study sample was 152 dogs (range, 74–451 dogs). Frailty was associated with increased mortality risk in five studies; in three studies [27, 29, 30] the relationship between frailty and mortality was not evaluated. Across studies, no clear male-female differences in frailty were reported. However, the relationship between gonad exposure and frailty was not evaluated in any of the previous dog studies. None of the eight studies reported years of lifetime gonad exposure, but instead data, if presented, were limited to gonad status (gonadectomized or intact) at the time of frailty scoring. Whether the prevalence of frailty differed on the basis of gonad status at time of frailty scoring was evaluated in only one study and was not significant [30]. Here, by capturing detailed information on age at gonadectomy in a cohort of dogs with a broad range of lifetime gonad exposure, we provide evidence for a strong relationship between gonad exposure and late-life robustness in both male and female dogs. Notably, no significant male-female differences were found after controlling for age at frailty scoring and gonad exposure. To replicate our main findings and to advance the study of sex differences in frailty, future research on frailty in companion dogs should report for males and females separately the age at gonadectomy (i.e., duration of lifetime gonad exposure) and reason for gonadectomy.

Most human clinical studies support the notion that, compared to men, women are usually more frail even though they live longer [7]. A meta-analysis of more than 37,000 subjects in five studies showed that females had higher FI values than males in any age group [72]. Similarly, a systematic review of more than 40,000 community dwelling adults > 65 years of age showed that the prevalence of frailty, measured by frailty phenotype, was significantly higher in women (9.6%) compared to only 5.2% in men [8]. The literature does contain a few studies that report male-female frailty patterns that deviate from this typical result [73, 74]. Some of the biological mechanisms that have been implicated in frailty development that might contribute to observed male-female differences include inflammation [75, 76], mitochondrial dysfunction [77], cellular senescence [78], and epigenetic changes [79]. It is widely accepted that several key physiological processes are sensitive to the influence of gonadal hormones. For example, estrogens promote skeletal muscle fiber synthesis and stimulate muscle repair [80], while testosterone exerts well-documented increases in muscle mass over the lifespan [81].

It is possible that observed sex differences in frailty may be affected by the method used to assess frailty. One of the proposed advances in the assessment of frailty is the use of tools that also capture laboratory or test-based measures, rather than relying solely on self-reported subjective health information [82]. Interestingly, in two separate large cohorts (The Irish Longitudinal Study of Aging, NHANES), laboratory-based FI values did not demonstrate the expected sex difference [9, 10]. In one study, although women had higher self-reported frailty, test-based measures of FI showed men had slightly higher frailty values than women [9]. In the other study, laboratory FI values were higher in women aged 20–39 years compared to men of the same age, but this pattern reversed in midlife, with men having higher FI values than women after age 60 years [10]. Taken together, it seems our understanding of sex differences in frailty, as well as their mechanistic underpinnings, could benefit greatly from additional longitudinal studies using frailty assessment tools that blend clinical and laboratory data at different ages throughout the life course.

In the current study, we focused on lifetime gonad exposure as a biological factor that might significantly contribute to male-female differences in frailty. However, it is well-established that non-biological, sociobehavioral influences may account for differences in frailty observed in men and women. Table 7 compiles a list of some of the sex- and gender-sensitive factors that may contribute to frailty differences in human studies, adapted from the review of Zeidan et al. [7]. Differences between men and women in health perception and the reporting of symptoms [44, 45, 83], engagement in caregiving [84, 85], community participation [86], religious activities [87, 88], and education level [89] have been linked to frailty, yet it remains unclear the extent to which the interaction of these factors contributes to the differences in frailty progression or improvement observed in men and women. Though a host of factors at play in human studies have been circumvented by our dog study design, dogs are social animals and therefore subject to interindividual sociobehavioral differences. Future work could assess whether particular sociobehavioral differences in dogs, such as household population density or degree of playfulness, impact late-life frailty in a sexually dimorphic manner. Clearly, further research across species on the sociobehavioral underpinnings of physical frailty is necessary to more adequately bridge this critical knowledge gap.

**Table 7:** Catalogue of sex- and gender-sensitive factors that may contribute to frailty in human studies, thereby obscuring evaluation of specific biological determinants of physical frailty, that can be side-stepped in dog studies (adapted from review of Zeidan et al. [7])

Sex- and Gender-Sensitive Factors	
Likelihood of seeking support from others	
Likelihood of maladaptive coping strategies	
Inclination to seek medical help and to follow through	
Tendency to downplay or ignore symptoms	
Differences in health perception	
Strength of social network	
Likelihood of engagement in caregiving	
Level of community participation	
Frequency of engagement in religious activities	
Likelihood of rating life events as less controllable	
Education levels	
Differences in motivations for exercise	
Response to new information on diet and health	
Smoking behavior	
Likelihood of experiencing sleep disturbances	

See ref 7 for sources and more detail

Alternative explanations for the absence of male-female frailty differences in the current study should be considered. We studied dogs in an attempt to remove some of the sociobehavioral influences that might be responsible for the male-female differences observed in humans. Our frailty scores in dogs, however, were based upon interviews with men and women, who may have reported deficits differently. Our analysis showed no difference in likelihood of late-life robustness among dogs whose frailty was reported by men versus women. Moreover, no significant male-female difference was present after adjusting the likelihood of late-life robustness for man versus woman reporting of dog frailty. It is possible that our use of a clinical FI, rather than frailty phenotype, was responsible for masking sex differences. However, human studies in the published literature have documented sex differences using either FI or frailty phenotype. Finally, we measured late-life frailty in extreme aged dogs in the EARS cohort. The relationship between sex and physical frailty may be age-sensitive. In one study in which women had higher lifetime FI values, frailty trajectories for the two sexes converged at extreme age, with curves crossing over at 94 years [90]. In another study, men had lower frailty values at younger ages, but accumulated deficits faster than women accounting for a crossover at approximately 85 years [91]. Because we compared FI values in males and females only at extreme age, our study design precluded gathering insights on sex-specific trajectories that could only be provided by longitudinal studies of deficit accumulation.

Our study has limitations. The relatively small size of our analytical sample precluded evaluation of robustness using a more severe cutpoint, such as FI quartile, and in some instances risk analyses yielded point estimates with broad confidence intervals. Information on age at gonadectomy was collected retrospectively and therefore subject to recall bias, though in more than 85% of cases the date of gonadectomy surgery could be confirmed through medical records. In this observational cohort, the key exposure variable, duration of lifetime gonad exposure, was not randomized. Accordingly, a subset of dogs might have been gonadectomized because of pre-existing health deficits (e.g., seizures, hip dysplasia, prostatitis) that may have potentially influenced lifetime health trajectory, thereby impacting the relationship between gonad removal and retention of late-life robustness. We addressed this possibility by conducting standardized interviews with pet owners to secure information on the reason for gonadectomy in each dog. We found no association between reason for gonadectomy and likelihood of late-life robustness. Moreover, the strong relationship between longer gonad exposure and increased likelihood of late-life robustness was not attenuated by whether or not pre-existing health deficits precipitated an owner’s decision for gonadectomy. It should be noted, however, that the elective decision by owners to remove gonads from companion dogs is made based upon multifaceted considerations that extend beyond animal health, that may relate to the pet owner and the environment in which the dog lives [25, 92, 93]. Our study of gonad exposure and robustness relied upon cross-sectional use of a clinical frailty index, EARS-FI. Therefore, we have not demonstrated that this clinical FI can be used to study longitudinal changes in frailty. Also, the possibility in females that the production of live offspring (i.e., parity) comes at a frailty cost [94, 95], thereby contributing to late-life deficit accumulation, was not evaluated in this study. Finally, the current work requires replication in dog studies that will show the extent to which our main findings are age-dependent or breed-dependent.

We studied dogs, not humans, in our evaluation of the relationship between HPG axis and development of frailty. Therefore, as in all preclinical studies [96, 97], it is not clear the extent to which the results are directly translatable to men or women. Because our study did not measure gonadal hormone levels and no dogs received hormone replacement, the results do not directly contribute to the development and refinement of functional anabolic or hormone-replacement therapies for frail older men or women. The cohort of North American Rottweiler dogs that reached extreme age that we studied represents a unique population. Therefore, the associations and effect sizes reported here may not be directly translatable to other Rottweiler populations or other dog breeds. The strength of the current study, however, resides in its use of a lifetime cohort of dogs to generate frailty scores in geriatric males and females with a broad range of lifetime gonad exposure, thereby applying a life course approach to studying the biological differences in physical frailty between males and females. There is clear precedent for dogs playing a translational role in endocrine research relevant to human health, including the discoveries of insulin [98] and androgen ablation for regression of prostate cancer [99, 100]. We believe the current work signals an important first step toward revealing the value of enlisting companion dogs in further investigations of the biology of sex differences in frailty.

## Conclusion

This research in companion dogs supports the notion that gonadal hormones exert an important impact on the retention of late-life robustness in both males and females. The work also points to the value of investigating the role early life events play in shaping trajectories of lifelong health. Such investigative approaches may prove instrumental in achieving the overarching goal of developing sex-specific anti-frailty strategies.

## Electronic supplementary material

Below is the link to the electronic supplementary material.

Supplementary Material 1

Supplementary Material 2

Supplementary Material 3

Supplementary Material 4

Supplementary Material 5

## Data Availability

The data generated or analyzed during this study are included in this published article and its Supplementary Information files. Further inquiries or requests regarding the data can be directed to the corresponding author.

## References

[1] Rockwood K, Blodgett JM, Theou O, Sun MH, Feridooni HA, Mitnitski A, et al. A frailty index based on deficit accumulation quantifies mortality risk in humans and in mice. Sci Rep. 2017;7(1):43068. 28220898 10.1038/srep43068 PMC5318852

[2] Rodríguez-Mañas L, Féart C, Mann G, Viña J, Chatterji S, Chodzko-Zajko W, et al. Searching for an operational definition of frailty: A Delphi method based consensus statement. The frailty operative definition-Consensus conference project. J Gerontol Ser A. 2013;68(1):62–7. 10.1093/gerona/gls119 PMC3598366 22511289

[3] Leng S, Chen X, Mao G. Frailty syndrome: an overview. Clin Interv Aging. 2014;9:433–41. 10.2147/CIA.S45300 PMC3964027 24672230

[4] Arosio B, Ferri E, Casati M, Mari D, Vitale G, Cesari M. The frailty index in centenarians and their offspring. Aging Clin Exp Res. 2019;31(11):1685–8. 31359370 10.1007/s40520-019-01283-7

[5] Searle SD, Mitnitski A, Gahbauer EA, Gill TM, Rockwood K. A standard procedure for creating a frailty index. BMC Geriatr. 2008;8(1):24. 18826625 10.1186/1471-2318-8-24 PMC2573877

[6] Hoogendijk EO, Theou O, Rockwood K, Onwuteaka-Philipsen BD, Deeg DJH, Huisman M. Development and validation of a frailty index in the longitudinal aging study Amsterdam. Aging Clin Exp Res. 2017;29(5):927–33. 27896796 10.1007/s40520-016-0689-0 PMC5589777

[7] Zeidan RS, McElroy T, Rathor L, Martenson MS, Lin Y, Mankowski RT. Sex differences in frailty among older adults. Exp Gerontol. 2023;184:112333. 37993077 10.1016/j.exger.2023.112333

[8] Collard RM, Boter H, Schoevers RA, Oude Voshaar RC. Prevalence of frailty in Community-Dwelling older persons: A systematic review. J Am Geriatr Soc. 2012;60(8):1487–92. 22881367 10.1111/j.1532-5415.2012.04054.x

[9] Theou O, O‘Connell MDL, King-Kallimanis BL, O’Halloran AM, Rockwood K, Kenny RA. Measuring frailty using self-report and test-based health measures. Age Ageing. 2015;44(3):471–7. 25687601 10.1093/ageing/afv010 PMC4411224

[10] Blodgett JM, Theou O, Mitnitski A, Howlett SE, Rockwood K. Associations between a laboratory frailty index and adverse health outcomes across age and sex. AGING Med. 2019;2(1):11–7. 10.1002/agm2.12055 PMC6880698 31942508

[11] Hubbard RE. Sex Differences in Frailty. In: Theou O, Rockwood K,Interdisciplinary Topics in Gerontology and Geriatrics [Internet]., Karger S. AG; 2015 [cited 2025 Feb 24]. pp. 41–53. Available from: https://karger.com/books/book/2976/chapter/5844140 10.1159/000381161 26301978

[12] Gordon E, Hubbard R. Physiological basis for sex differences in frailty. Curr Opin Physiol. 2018;6:10–5.

[13] Gordon EH, Hubbard RE. Differences in frailty in older men and women. Med J Aust. 2020;212(4):183–8. 31886526 10.5694/mja2.50466

[14] Kane AE, Howlett SE. Sex differences in frailty: comparisons between humans and preclinical models. Mech Ageing Dev. 2021;198:111546. 34324923 10.1016/j.mad.2021.111546

[15] Oksuzyan A, Juel K, Vaupel JW, Christensen K. Men: good health and high mortality. Sex differences in health and aging. Aging Clin Exp Res. 2008;20(2):91–102. 18431075 10.1007/bf03324754 PMC3629373

[16] Gender Equality Index 2021: Health [Internet]. Available from: https://eige.europa.eu/publications-resources/toolkits-guides/gender-equality-index-2021-report/men-are-more-likely-perceive-their-health-good?language_content_

[17] Parks RJ, Fares E, MacDonald JK, Ernst MC, Sinal CJ, Rockwood K, et al. A procedure for creating a frailty index based on deficit accumulation in aging mice. J Gerontol Ser A. 2012;67A(3):217–27. 10.1093/gerona/glr193 22021390

[18] Kane AE, Keller KM, Heinze-Milne S, Grandy SA, Howlett SE. A murine frailty index based on clinical and laboratory measurements: links between frailty and Pro-inflammatory cytokines differ in a Sex-Specific manner. J Gerontol Ser A. 2019;74(3):275–82. 10.1093/gerona/gly117 PMC6376103 29788087

[19] Waters DJ. Aging research 2011: exploring the pet dog paradigm. ILAR J. 2011;52(1):97–105. 21411862 10.1093/ilar.52.1.97

[20] Head E. A canine model of human aging and alzheimer’s disease. Biochim Biophys Acta BBA - Mol Basis Dis. 2013;1832(9):1384–9. 10.1016/j.bbadis.2013.03.016 PMC3937962 23528711

[21] Gilmore KM, Greer KA. Why is the dog an ideal model for aging research? Exp Gerontol. 2015;71:14–20. 26325590 10.1016/j.exger.2015.08.008

[22] Mazzatenta A, Carluccio A, Robbe D, Giulio CD, Cellerino A. The companion dog as a unique translational model for aging. Semin Cell Dev Biol. 2017;70:141–53. 28803893 10.1016/j.semcdb.2017.08.024

[23] Hoffman JM, Creevy KE, Franks A, O’Neill DG, Promislow DEL. The companion dog as a model for human aging and mortality. Aging Cell. 2018;17(3):e12737. 29457329 10.1111/acel.12737 PMC5946068

[24] Creevy KE, Akey JM, Kaeberlein M, Promislow DEL, The Dog Aging Project Consortium, Barnett BG, et al. An open science study of ageing in companion dogs. Nature. 2022;602(7895):51–7. 35110758 10.1038/s41586-021-04282-9 PMC8940555

[25] Zink MC, Farhoody P, Elser SE, Ruffini LD, Gibbons TA, Rieger RH. Evaluation of the risk and age of onset of cancer and behavioral disorders in gonadectomized Vizslas. J Am Vet Med Assoc. 2014;244(3):309–19. 24432963 10.2460/javma.244.3.309

[26] Banzato T, Franzo G, Di Maggio R, Nicoletto E, Burti S, Cesari M, et al. A frailty index based on clinical data to quantify mortality risk in dogs. Sci Rep. 2019;9(1):16749. 31727920 10.1038/s41598-019-52585-9 PMC6856105

[27] Chen FL, Ullal TV, Graves JL, Ratcliff ER, Naka A, McKenzie B, et al. Evaluating instruments for assessing healthspan: a multi-center cross-sectional study on health-related quality of life (HRQL) and frailty in the companion dog. GeroScience. 2023;45(4):2089–108. 36781597 10.1007/s11357-023-00744-2 PMC10651603

[28] Waters DJ, Maras AH, Fu R, Carrillo AE, Chiang EC, Suckow CL. Frailty and mortality risk among dogs with extreme longevity: development and predictive validity of a clinical frailty index in the Exceptional Aging in Rottweilers Study. Animals. 2024;14(24):3651. 39765555 10.3390/ani14243651 PMC11672423

[29] McKenzie B, Peloquin M, Graves JL, Chen F, Tovar A, Carttar TA, et al. Changes in insulin, adiponectin and lipid concentrations with age are associated with frailty and reduced quality of life in dogs. Sci Rep. 2025;15(1):5380. 39948141 10.1038/s41598-025-89923-z PMC11825863

[30] Blanchard T, Mugnier A, Déjean S, Priymenko N, Meynadier A. Exploring frailty in apparently healthy senior dogs: a cross-sectional study. BMC Vet Res. 2024;20(1):436. 39342207 10.1186/s12917-024-04296-1 PMC11438228

[31] Russell KJ, Mondino A, Fefer G, Griffith E, Saker K, Gruen ME, et al. Establishing a clinically applicable frailty phenotype screening tool for aging dogs. Front Vet Sci. 2024;11:1335463. 39391218 10.3389/fvets.2024.1335463 PMC11465091

[32] Hua J, Hoummady S, Muller C, Pouchelon JL, Blondot M, Gilbert C, et al. Assessment of frailty in aged dogs. Am J Vet Res. 2016;77(12):1357–65. 27901392 10.2460/ajvr.77.12.1357

[33] Lemaréchal R, Hoummady S, Barthélémy I, Muller C, Hua J, Gilbert C et al. Canine Model of Human Frailty: Adaptation of a Frailty Phenotype in Older Dogs. Le Couteur D, editor. J Gerontol Ser A. 2023;78(8):1355–63. 10.1093/gerona/glad006 36617213

[34] Peng X, Hou L, Zhao Y, Lin T, Wang H, Gao L, et al. Frailty and testosterone level in older adults: a systematic review and meta-analysis. Eur Geriatr Med. 2022;13(3):663–73. 35107811 10.1007/s41999-022-00614-8

[35] Chu W, Lynskey N, Iain-Ross J, Pell JP, Sattar N, Ho FK, et al. Identifying the biomarker profile of Pre-Frail and frail people: A Cross-Sectional analysis from UK biobank. Int J Environ Res Public Health. 2023;20(3):2421. 36767787 10.3390/ijerph20032421 PMC9915970

[36] Kojima G, Taniguchi Y, Ogawa K, Aoyama R, Urano T. Age at menopause is negatively associated with frailty: A systematic review and meta-analysis. Maturitas. 2022;165:94–9. 35940027 10.1016/j.maturitas.2022.07.012

[37] Kojima G, Taniguchi Y, Aoyama R, Urano T. Earlier menopause is associated with higher risk of incident frailty in community-dwelling older women in England. J Am Geriatr Soc. 2022;70(9):2602–9. 35546044 10.1111/jgs.17838

[38] Verschoor CP, Tamim H. Frailty is inversely related to age at menopause and elevated in women who have had a hysterectomy: an analysis of the Canadian longitudinal study on aging. J Gerontol Ser A. 2019;74(5):675–82. 10.1093/gerona/gly092 PMC6477649 29688443

[39] Ho VWT, Chua KY, Song X, Jin A, Koh WP. Reproductive factors and risk of physical frailty among Chinese women living in Singapore. J Nutr Health Aging. 2024;28(6):100226.38593634 10.1016/j.jnha.2024.100226

[40] Simon KE, Russell K, Mondino A, Yang CC, Case BC, Anderson Z, et al. A randomized, controlled clinical trial demonstrates improved owner-assessed cognitive function in senior dogs receiving a senolytic and NAD + precursor combination. Sci Rep. 2024;14(1):12399. 38811634 10.1038/s41598-024-63031-w PMC11137034

[41] O’Neill DG, Seah WY, Church DB, Brodbelt DC. Rottweilers under primary veterinary care in the UK: demography, mortality and disorders. Canine Genet Epidemiol. 2017;4(1):13. 29201384 10.1186/s40575-017-0051-7 PMC5698930

[42] Waters DJ, Kengeri SS, Maras AH, Suckow CL, Chiang EC. Life course analysis of the impact of mammary cancer and pyometra on age-anchored life expectancy in female rottweilers: implications for envisioning ovary conservation as a strategy to promote healthy longevity in pet dogs. Vet J. 2017;224:25–37. 28697872 10.1016/j.tvjl.2017.05.006

[43] Waters DJ, Fu R, Carrillo AE, Chiang EC, Maras AH, Kengeri SS, et al. Correlates of estimated lifetime cruciate ligament survival inform potential rupture risk reduction strategies: findings from the Exceptional Aging in Rottweilers Study. Sci Rep. 2023;13(1):13920. 37626101 10.1038/s41598-023-39288-y PMC10457323

[44] Banks I. No man’s land: men, illness, and the NHS. BMJ. 2001;323(7320):1058–60. 11691768 10.1136/bmj.323.7320.1058 PMC1121551

[45] Lee KS, Feltner FJ, Bailey AL, Lennie TA, Chung ML, Smalls BL, et al. The relationship between psychological States and health perception in individuals at risk for cardiovascular disease. Psychol Res Behav Manag. 2019;12:317–24. 31191053 10.2147/PRBM.S198280 PMC6520523

[46] Ukraintseva S, Yashin AI, Arbeev KG. Resilience versus robustness in aging. J Gerontol Biol Sci Med Sci. 2016;71(11):1533–4. 10.1093/gerona/glw083 PMC6279216 27146372

[47] Wallace LMK, Theou O, Godin J, Andrew MK, Bennett DA, Rockwood K. Investigation of frailty as a moderator of the relationship between neuropathology and dementia in alzheimer’s disease: a cross-sectional analysis of data from the rush memory and aging project. Lancet Neurol. 2019;18(2):177–84. 30663607 10.1016/S1474-4422(18)30371-5 PMC11062500

[48] Ravindrarajah R, Hazra NC, Hamada S, Charlton J, Jackson SHD, Dregan A, et al. Systolic blood pressure trajectory, frailty, and All-Cause mortality > 80 years of age: cohort study using electronic health records. Circulation. 2017;135(24):2357–68. 28432148 10.1161/CIRCULATIONAHA.116.026687 PMC5472195

[49] Shaw BH, Borrel D, Sabbaghan K, Kum C, Yang Y, Robinovitch SN, et al. Relationships between orthostatic hypotension, frailty, falling and mortality in elderly care home residents. BMC Geriatr. 2019;19(1):80. 30866845 10.1186/s12877-019-1082-6 PMC6415493

[50] Clegg A, Bates C, Young J, Ryan R, Nichols L, Ann Teale E, et al. Development and validation of an electronic frailty index using routine primary care electronic health record data. Age Ageing. 2016;45(3):353–60. 26944937 10.1093/ageing/afw039 PMC4846793

[51] Rockwood K, Mitnitski A. Limits to deficit accumulation in elderly people. Mech Ageing Dev. 2006;127(5):494–6. 16487992 10.1016/j.mad.2006.01.002

[52] Canevelli M, Jackson-Tarlton C, Rockwood K. Frailty for neurologists: perspectives on how frailty influences care planning. Lancet Neurol. 2024;23(11):1147–57. 10.1016/S1474-4422(24)00291-6 39276779

[53] Gordon EH, Reid N, Khetani IS, Hubbard RE. How frail is frail? A systematic scoping review and synthesis of high impact studies. BMC Geriatr. 2021;21(1):719. 34922490 10.1186/s12877-021-02671-3 PMC8684089

[54] Fletcher JA, Logan B, Reid N, Gordon EH, Ladwa R, Hubbard RE. How frail is frail in oncology studies? A scoping review. BMC Cancer. 2023;23(1):498. 37268891 10.1186/s12885-023-10933-z PMC10236730

[55] Bhasin S. The brave new world of Function-Promoting anabolic therapies: testosterone and frailty. J Clin Endocrinol Metab. 2010;95(2):509–11. 20133471 10.1210/jc.2009-2550

[56] Van Den Beld AW, Huhtaniemi IT, Pettersson KSL, Pols HAP, Grobbee DE, De Jong FH, et al. Luteinizing hormone and different genetic variants, as indicators of frailty in healthy elderly men. J Clin Endocrinol Metab. 1999;84(4):1334–9. 10199775 10.1210/jcem.84.4.5616

[57] Tajar A, O’Connell MDL, Mitnitski AB, O’Neill TW, Searle SD, Huhtaniemi IT, et al. Frailty in relation to variations in hormone levels of the Hypothalamic-Pituitary-Testicular Axis in older men: results from the European male aging study: REPRODUCTIVE HORMONES AND FRAILTY. J Am Geriatr Soc. 2011;59(5):814–21. 21568952 10.1111/j.1532-5415.2011.03398.x

[58] Mohr BA, Bhasin S, Kupelian V, Araujo AB, O’Donnell AB, McKinlay JB. Testosterone, sex Hormone–Binding globulin, and frailty in older men. J Am Geriatr Soc. 2007;55(4):548–55. 17397433 10.1111/j.1532-5415.2007.01121.x

[59] Cawthon PM, Ensrud KE, Laughlin GA, Cauley JA, Dam TTL, Barrett-Connor E, et al. Sex hormones and frailty in older men: the osteoporotic fractures in men (MrOS) study. J Clin Endocrinol Metab. 2009;94(10):3806–15. 19737923 10.1210/jc.2009-0417 PMC2758722

[60] Cappola AR, Xue QL, Fried LP. Multiple hormonal deficiencies in anabolic hormones are found in frail older women: the women’s health and aging studies. J Gerontol Biol Sci Med Sci. 2009;64A(2):243–8. 10.1093/gerona/gln026 PMC2655016 19182229

[61] Carcaillon L, Blanco C, Alonso-Bouzón C, Alfaro-Acha A, Garcia-García FJ, Rodriguez-Mañas L. Sex Differences in the Association between Serum Levels of Testosterone and Frailty in an Elderly Population: The Toledo Study for Healthy Aging. Vina J, editor. PLoS ONE. 2012;7(3):e32401. 10.1371/journal.pone.0032401 PMC3293806 22403651

[62] Eichholzer M, Barbir A, Basaria S, Dobs AS, Feinleib M, Guallar E, et al. Serum sex steroid hormones and frailty in older American men of the third National health and nutrition examination survey (NHANES III). Aging Male. 2012;15(4):208–15. 22822787 10.3109/13685538.2012.705366 PMC4581525

[63] Serra-Prat M, Papiol M, Vico J, Palomera E, Sist X, Cabré M. Factors associated with frailty in community-dwelling elderly population. A cross-sectional study. Eur Geriatr Med. 2016;7(6):531–7.

[64] Huang G, Coviello A, LaValley MP, Ensrud KE, Cauley JA, Cawthon PM, et al. Surgical menopause and frailty risk in Community-Dwelling older women: study of osteoporotic fractures. J Am Geriatr Soc. 2018;66(11):2172–7. 30251302 10.1111/jgs.15505 PMC6292428

[65] Swiecicka A, Eendebak RJAH, Lunt M, O’Neill TW, Bartfai G, Casanueva FF, et al. Reproductive hormone levels predict changes in frailty status in Community-Dwelling older men: European male ageing study prospective data. J Clin Endocrinol Metab. 2018;103(2):701–9. 29186457 10.1210/jc.2017-01172 PMC5800832

[66] Chiang JM, Kaysen GA, Segal M, Chertow GM, Delgado C, Johansen KL. Low testosterone is associated with frailty, muscle wasting and physical dysfunction among men receiving hemodialysis: a longitudinal analysis. Nephrol Dial Transpl. 2019;34(5):802–10. 10.1093/ndt/gfy252 PMC6503299 30085235

[67] Travison TG, Nguyen AH, Naganathan V, Stanaway FF, Blyth FM, Cumming RG, et al. Changes in reproductive hormone concentrations predict the prevalence and progression of the frailty syndrome in older men: the concord health and ageing in men project. J Clin Endocrinol Metab. 2011;96(8):2464–74. 21677041 10.1210/jc.2011-0143

[68] Connolly MJ, Kerse N, Wilkinson T, Menzies O, Rolleston A, Chong YH, et al. Testosterone in advance age: a new Zealand longitudinal cohort study: life and living in advanced age (Te Puāwaitanga o Ngā Tapuwae Kia Ora Tonu). BMJ Open. 2017;7(11):e016572. 29133315 10.1136/bmjopen-2017-016572 PMC5695316

[69] Hyde Z, Flicker L, Almeida OP, Hankey GJ, McCaul KA, Chubb SAP, et al. Low free testosterone predicts frailty in older men: the health in men study. J Clin Endocrinol Metab. 2010;95(7):3165–72. 20410223 10.1210/jc.2009-2754

[70] Heinze-Milne SD, Banga S, Godin J, Howlett SE. Serum testosterone concentrations are not associated with frailty in naturally ageing and testosterone-deficient older C57Bl/6 mice. Mech Ageing Dev. 2022;203:111638. 35124093 10.1016/j.mad.2022.111638

[71] Rocca WA, Gazzuola Rocca L, Smith CY, Grossardt BR, Faubion SS, Shuster LT, et al. Loss of ovarian hormones and accelerated somatic and mental aging. Physiology. 2018;33(6):374–83. 30303778 10.1152/physiol.00024.2018 PMC6734081

[72] Gordon EH, Peel NM, Samanta M, Theou O, Howlett SE, Hubbard RE. Sex differences in frailty: A systematic review and meta-analysis. Exp Gerontol. 2017;89:30–40. 28043934 10.1016/j.exger.2016.12.021

[73] Bartley MM, Geda YE, Christianson TJH, Shane Pankratz V, Roberts RO, Petersen RC. Frailty and mortality outcomes in cognitively normal older people: sex differences in a Population-Based study. J Am Geriatr Soc. 2016;64(1):132–7. 26782862 10.1111/jgs.13821 PMC4721254

[74] Saum KU, Dieffenbach AK, Müller H, Holleczek B, Hauer K, Brenner H. Frailty prevalence and 10-year survival in community-dwelling older adults: results from the ESTHER cohort study. Eur J Epidemiol. 2014;29(3):171–9. 24671603 10.1007/s10654-014-9891-6

[75] Samson LD, Boots AMH, Ferreira JA, Picavet HSJ, De Rond LGH, De Zeeuw-Brouwer M et al. lène,. In-depth immune cellular profiling reveals sex-specific associations with frailty. Immun Ageing. 2020;17(1):20. 10.1186/s12979-020-00191-z PMC7310472 32582361

[76] Van Sleen Y, Shetty SA, Van Der Heiden M, Venema MCA, Gutiérrez-Melo N, Toonen EJM, et al. Frailty is related to serum inflammageing markers: results from the VITAL study. Immun Ageing. 2023;20(1):68. 38012652 10.1186/s12979-023-00391-3 PMC10680197

[77] Kristensen TN, Loeschcke V, Tan Q, Pertoldi C, Mengel-From J. Sex and age specific reduction in stress resistance and mitochondrial DNA copy number in Drosophila melanogaster. Sci Rep. 2019;9(1):12305. 31444377 10.1038/s41598-019-48752-7 PMC6707197

[78] Taylor JA, Greenhaff PL, Bartlett DB, Jackson TA, Duggal NA, Lord JM. Multisystem physiological perspective of human frailty and its modulation by physical activity. Physiol Rev. 2023;103(2):1137–91. 36239451 10.1152/physrev.00037.2021 PMC9886361

[79] Fischer KE, Riddle NC. Sex differences in aging: genomic instability. J Gerontol Ser A. 2018;73(2):166–74. 10.1093/gerona/glx105 PMC5861920 28575157

[80] Enns DL, Tiidus PM. The influence of Estrogen on skeletal muscle: sex matters. Sports Med. 2010;40(1):41–58. 20020786 10.2165/11319760-000000000-00000

[81] Mouser JG, Loprinzi PD, Loenneke JP. The association between physiologic testosterone levels, lean mass, and fat mass in a nationally representative sample of men in the united States. Steroids. 2016;115:62–6. 27543675 10.1016/j.steroids.2016.08.009

[82] Sapp DG, Cormier BM, Rockwood K, Howlett SE, Heinze SS. The frailty index based on laboratory test data as a tool to investigate the impact of frailty on health outcomes: a systematic review and meta-analysis. Age Ageing. 2023;52(1):afac309. 36626319 10.1093/ageing/afac309 PMC9831271

[83] Yiğitalp G, Bayram Değer V, Çifçi S. Health literacy, health perception and related factors among different ethnic groups: a cross-sectional study in southeastern Turkey. BMC Public Health. 2021;21(1):1109. 34112137 10.1186/s12889-021-11119-7 PMC8194111

[84] Caputo J, Pavalko EK, Hardy MA. The Long-Term effects of caregiving on women’s health and mortality. J Marriage Fam. 2016;78(5):1382–98. 27795579 10.1111/jomf.12332 PMC5079527

[85] Wennberg AM, Anderson LR, Chen-Edinboro LP, Cagnin A, Pini L. Positive Aspects of Caregiving Are Associated With Lower Risk of Frailty and Sleep Disruption in the National Study of Caregiving. Savla JT, editor. Innov Aging. 2022;6(7):igac058. 10.1093/geroni/igac058 PMC9579720 36267323

[86] Chen L, Zhang Z. Community participation and subjective Well-Being of older adults: the roles of sense of community and neuroticism. Int J Environ Res Public Health. 2022;19(6):3261. 35328950 10.3390/ijerph19063261 PMC8953512

[87] Kim A, Yi E, Kim J, Kim M. A study on the influence of social leisure activities on the progression to the stage of frailty in Korean seniors. Int J Environ Res Public Health. 2020;17(23):8909. 33266136 10.3390/ijerph17238909 PMC7731322

[88] Naud D, Généreux M, Bruneau JF, Alauzet A, Levasseur M. Social participation in older women and men: differences in community activities and barriers according to region and population size in Canada. BMC Public Health. 2019;19(1):1124. 31420061 10.1186/s12889-019-7462-1 PMC6697934

[89] Hoogendijk EO, Van Hout HPJ, Heymans MW, Van Der Horst HE, Frijters DHM, Van Broese MI, et al. Explaining the association between educational level and frailty in older adults: results from a 13-year longitudinal study in the Netherlands. Ann Epidemiol. 2014;24(7):538–e5442. 24935466 10.1016/j.annepidem.2014.05.002

[90] Mitnitski AB, Mogilner AJ, MacKnight C, Rockwood K. The accumulation of deficits with age and possible invariants of aging. Sci World J. 2002;2:1816–22. 10.1100/tsw.2002.861 PMC6009575 12806172

[91] Kulminski A, Yashin A, Ukraintseva S, Akushevich I, Arbeev K, Land K, et al. Accumulation of health disorders as a systemic measure of aging: findings from the NLTCS data. Mech Ageing Dev. 2006;127(11):840–8. 16978683 10.1016/j.mad.2006.08.005 PMC1764645

[92] Kutzler MA. Possible relationship between Long-Term adverse health effects of Gonad-Removing surgical sterilization and luteinizing hormone in dogs. Animals. 2020;10(4):599. 32244716 10.3390/ani10040599 PMC7222805

[93] Zink C. Spay-neuter considerations to maximize health [Internet]. 2017. (Innovative Veterinary Care). Available from: https://ivcjournal.com/ spay- neuter- consi derat ions/.

[94] Gordon EH, Peel NM, Chatfield MD, Lang IA, Hubbard RE. Frailty: A cost incurred by reproduction? Sci Rep. 2020;10(1):10139. 32576951 10.1038/s41598-020-67009-2 PMC7311439

[95] Chang T, Zhao Z, Liu X, Zhang Y, Liu X, Zhang Y, et al. The association between reproductive factors and frailty risk: a population-based analysis from the UK biobank. BMC Public Health. 2025;25(1):762. 39994608 10.1186/s12889-025-22012-y PMC11853312

[96] Perazza LR, Brown-Borg HM, Thompson LV. Physiological Systems in Promoting Frailty. In: Prakash YS, editor. Comprehensive Physiology [Internet]. 1st ed. Wiley; 2022 [cited 2025 Mar 15]. pp. 3575–620. Available from: https://onlinelibrary.wiley.com/doi/10.1002/cphy.c210034 10.1002/cphy.c210034 PMC9531553 35578945

[97] Bartolomucci A, Kane AE, Gaydosh L, Razzoli M, McCoy BM, Ehninger D et al. Animal Models Relevant for Geroscience: Current Trends and Future Perspectives in Biomarkers, and Measures of Biological Aging. Duque G, editor. J Gerontol A Biol Sci Med Sci. 2024;79(9):glae135. 10.1093/gerona/glae135 PMC11316208 39126297

[98] Bliss M. The discovery of insulin. Toronto, Canada: University of Toronto; 1982.

[99] Huggins C, Clark PJ. Quantitative studies of prostatic secretion: II. The effect of castration and of Estrogen injection on the normal and on the hyperplastic prostate glands of dogs. J Exp Med. 1940;72(6):747–62. 19871058 10.1084/jem.72.6.747 PMC2135039

[100] Huggins C, Hodges CV. Studies on prostatic cancer. I. The effect of castration, of Estrogen and of androgen injection on serum phosphatases in metastatic carcinoma of the prostate. J Urol. 1941;1(4):293–7. 10.1016/s0022-5347(05)64820-3 12050481

