# Supplementary material for: Applying a life course approach to elucidate the biology of sex differences in frailty: early-life gonadectomy diminishes late-life robustness in male and female dogs in the Exceptional Aging in Rottweilers Study
Source: Biol Sex Differ. 2025 Jul 16;16:52. doi: 10.1186/s13293-025-00735-2 (PMC12265119; doi:10.1186/s13293-025-00735-2)
Supplement: Supplementary file 1 — Supplementary Material 1 [file 13293_2025_735_MOESM1_ESM.docx]

| **Supplementary Table 1** Re-evaluation of the likelihood of late-life robustness associated with duration of lifetime gonad exposure in 87 males using same gonad exposure cutpoint (5.5 years) used in analysis of females | | |
| --- | --- | --- |
|  | ***Adjusted OR (95% CI)** | ***p*-value** |
| Duration of  Lifetime Gonad Exposure |  |  |
| < 2 years | 1.0 (ref) |  |
| 2.0 – 5.5 years | 5.12 (0.50-52.60) | 0.17 |
| > 5.5 years | 9.76 (1.15-83.11) | 0.04 |
| Estimated likelihood of late-life robustness in males associated with duration of gonad exposure (i.e., duration of intact HPG axis) generated using logistic regression is shown as an adjusted odds ratio (OR) and 95% confidence interval (95%CI). Adjusted ORs are adjusted for age at frailty scoring, body condition, birth cohort, and pet owner reporting frailty. Age at frailty scoring was treated as a continuous variable (years). To assess duration of intact HPG axis, male dogs were segregated into three different gonad exposure groups based on age at gonadectomy (see text). Overweight body condition refers to overweight after seven years of age based on owner report. Late-life robustness was defined as frailty index values within the lowest tertile of the study population (n=222). ref = reference group. | | |
