# Supplementary material for: Applying a life course approach to elucidate the biology of sex differences in frailty: early-life gonadectomy diminishes late-life robustness in male and female dogs in the Exceptional Aging in Rottweilers Study
Source: Biol Sex Differ. 2025 Jul 16;16:52. doi: 10.1186/s13293-025-00735-2 (PMC12265119; doi:10.1186/s13293-025-00735-2)
Supplement: Supplementary file 2 — Supplementary Material 2 [file 13293_2025_735_MOESM2_ESM.docx]

| **Supplementary Table 2** Multivariate odds ratios (OR) for likelihood of late-life robustness associated with duration of lifetime gonad exposure and other risk variables in 84 males in which reason for gonadectomy was available | | |
| --- | --- | --- |
|  | **Multivariate OR**  **(95% CI)** | ***p*-value** |
| Age at Frailty Scoring | 1.01 (0.94-1.09) | 0.76 |
| Duration of  Lifetime Gonad Exposure |  |  |
| < 2 years | 1.0 (ref) |  |
| 2.0 – 9.8 years | 5.8 (0.68-53.31) | 0.12 |
| > 9.8 years | 10.63 (0.12-93.60) | 0.03 |
| Body Condition |  |  |
| Not overweight | 1.0 (ref) |  |
| Overweight | 0.73 (0.15-3.47) | 0.69 |
| Birth Cohort |  |  |
| Early | 1.0 (ref) |  |
| Late | 2.52 (0.83-7.68) | 0.11 |
| Pet Owner Reporting  Dog Frailty |  |  |
| Woman | 1.0 (ref) |  |
| Man | 0.44 (0.08-2.54) | 0.36 |
| Reason for Gonadectomy |  |  |
| No Deficit | 1.0 (ref) |  |
| Deficit | 0.54 (0.13-2.18) | 0.39 |
| Estimated likelihood of late-life robustness in males associated with duration of gonad exposure (i.e., duration of intact HPG axis) and other exposure variables generated using multivariate logistic regression is shown as an odds ratio (OR) and 95% confidence interval (95%CI). Multivariate ORs are calculated for each variable with all six variables in the multivariate analysis. Age at frailty scoring was treated as a continuous variable (years). To assess duration of intact HPG axis, male dogs were segregated into three different gonad exposure groups based on age at gonadectomy (see text). Overweight body condition refers to overweight after seven years of age based on owner report. Dogs were dichotomized into two groups on the basis of reason for gonadectomy: pre-existing health deficit (Deficit) or other reasons for gonadectomy (No Deficit). Odds ratio for the group with pre-existing Deficit as reason for gonadectomy are reported, with the No Deficit group as reference (ref) group. Late-life robustness was defined as frailty index values within the lowest tertile of the study population (n=222). | | |
