# Supplementary material for: Applying a life course approach to elucidate the biology of sex differences in frailty: early-life gonadectomy diminishes late-life robustness in male and female dogs in the Exceptional Aging in Rottweilers Study
Source: Biol Sex Differ. 2025 Jul 16;16:52. doi: 10.1186/s13293-025-00735-2 (PMC12265119; doi:10.1186/s13293-025-00735-2)
Supplement: Supplementary file 4 — Supplementary Material 4 [file 13293_2025_735_MOESM4_ESM.docx]

| **Supplementary Table 4** Comparison of male-female differences in unadjusted and age-adjusted odds ratios (OR) for the likelihood of late-life robustness in 222 dogs stratified into three categories of lifetime gonad exposure using 5.5 years as cutpoint in both sexes | | | | | |
| --- | --- | --- | --- | --- | --- |
| **Gonad Exposure Category** | **Sex** | **Unadjusted OR (95% CI)** | ***p*-value** | **Age-Adjusted OR (95% CI)** | ***p*-value** |
| Low*  <2 years | Males | 1.0 (ref) |  | 1.0 (ref) |  |
|  | Females | 4.03 (0.46-35.30) | 0.21 | 3.91 (0.44-34.58) | 0.22 |
|  |  |  |  |  |  |
| Middle**  2 – 5.5 years | Males | 1.0 (ref) |  | 1.0 (ref) |  |
|  | Females | 1.22 (0.37-4.02) | 0.74 | 1.27 (0.38-4.22) | 0.69 |
|  |  |  |  |  |  |
| High***  >5.5 years | Males | 1.0 (ref) |  | 1.0 (ref) |  |
|  | Females | 0.96 (0.42-2.20) | 0.92 | 1.01 (0.43-2.36) | 0.99 |
| Late-life robustness defined as frailty index values within the lowest tertile of the study population (n=222). Unadjusted and age-adjusted odds ratios (OR) and 95% confidence interval (95%CI) for each lifetime gonad exposure category reported for females using logistic regression. Males serve as the reference (ref) group (OR=1.0) for each gonad exposure category.  *Low lifetime gonad exposure group (n=60) consisting of 20 males with exposure <2 years, 40 females with exposure <2 years  **Middle lifetime gonad exposure group (n=68) consisting of 19 males with exposure 2 – 5.5 years, 49 females with exposure 2 – 5.5 years  ***High lifetime gonad exposure group (n=94) consisting of 48 males with exposure >5.5 years, 46 females with exposure >5.5 years | | | | | |
