# Supplementary figures and images for: Applying a life course approach to elucidate the biology of sex differences in frailty: early-life gonadectomy diminishes late-life robustness in male and female dogs in the Exceptional Aging in Rottweilers Study
Source: Biol Sex Differ. 2025 Jul 16;16:52. doi: 10.1186/s13293-025-00735-2 (PMC12265119; doi:10.1186/s13293-025-00735-2)

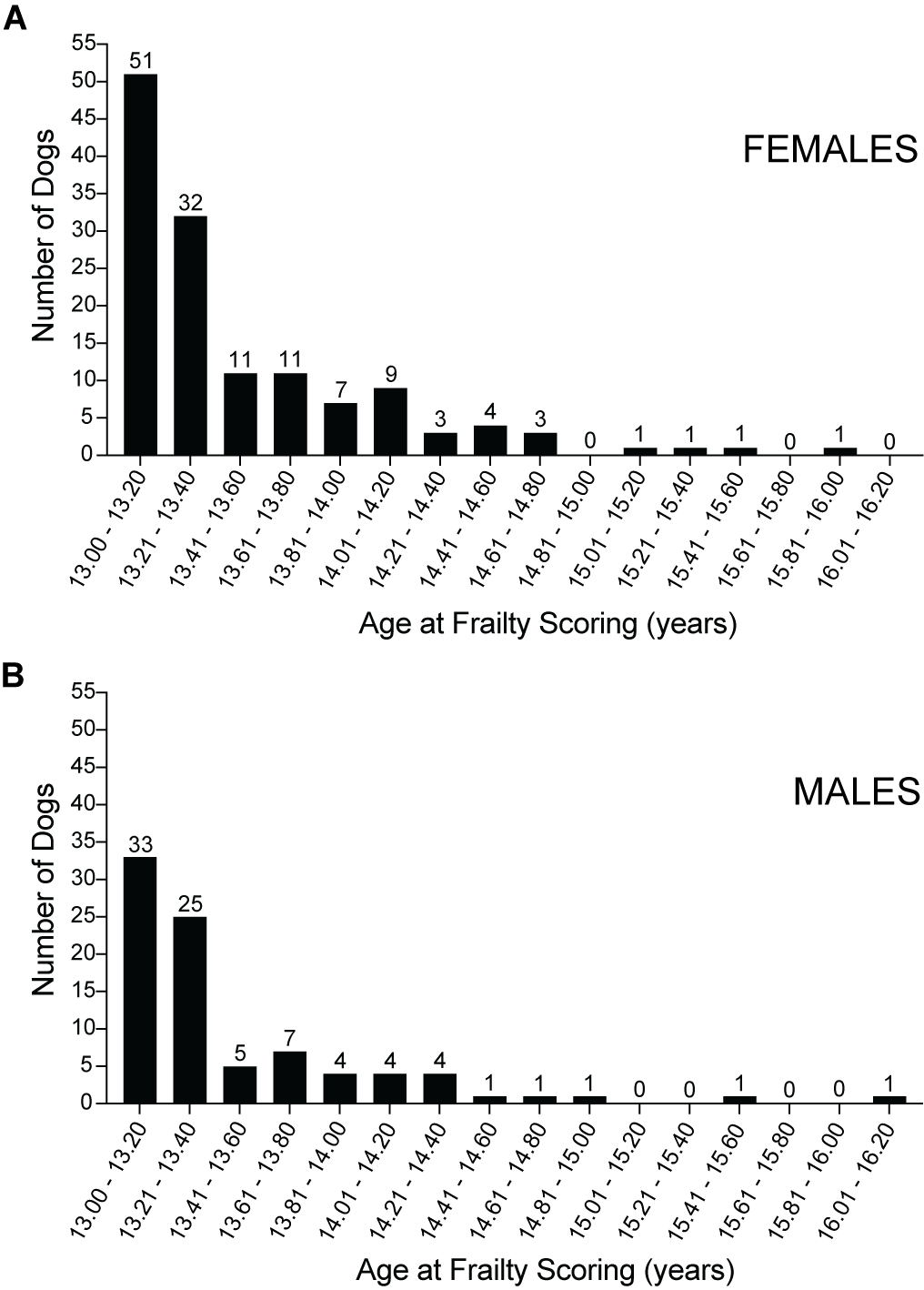

Supplement: Supplementary file 5 — Supplementary Material 5 [file 13293_2025_735_MOESM5_ESM.png]
